# Prevalence and predictors of postpartum depression by HIV status and timing of HIV diagnosis in Gauteng, South Africa

**DOI:** 10.1371/journal.pone.0214849

**Published:** 2019-04-04

**Authors:** Idah Mokhele, Cornelius Nattey, Nelly Jinga, Constance Mongwenyana, Matthew P. Fox, Dorina Onoya

**Affiliations:** 1 Health Economics and Epidemiology Research Office, Department of Internal Medicine, School of Clinical Medicine, Faculty of Health Sciences, University of the Witwatersrand, Johannesburg, South Africa; 2 Department of Global Health, Boston University School of Public Health, Boston, Massachusetts, United States of America; 3 Department of Epidemiology, Boston University School of Public Health, Boston, Massachusetts, United States of America; The Ohio State University, UNITED STATES

## Abstract

**Background:**

Postpartum depression (PPD) is a common mental health condition that can compromise the quality of life and functional capacity of mothers and cause health and developmental problems in children born to affected mothers.

**Objectives:**

We set out to measure the prevalence of PPD comparing postpartum HIV-1 infected women with pre-pregnancy HIV care experience, newly diagnosed (in latest pregnancy) HIV-1 infected women and HIV negative women, and to identify predictors of major PPD among these women in a peri-urban clinic in South Africa.

**Methods:**

We conducted a cross-sectional survey of 1151 adult (≥18 years) postpartum HIV-1 infected (690) and HIV negative (461) women who delivered up to 30 days before study enrolment, interviewed after their first post-natal visit (3–6 days post- delivery) at Midwife Obstetric Units in Gauteng, South Africa. PPD was categorised into no depression (CES-D 10 total score <5), low to medium depression (CES-D 10 total score ≥5 and <10) and major depressive symptoms (CES-D 10 total score≥10). We used ordered logistic regression to identify predictors of postpartum depression and report adjusted odds ratio (aOR) and 95% confidence intervals (CIs).

**Results:**

Overall 288 (25.0%) women screened positive for postpartum depression, a total of 168 (14.6%) women had low to medium PPD and 120 (10.4%) had major PPD. A higher proportion of HIV negative women experienced PPD, 129/461 (28.0%) among HIV negative vs. 159/690 (23.0%) among HIV-1 infected. Among HIV positive women, there was no meaningful difference in PPD between newly HIV diagnosed and those diagnosed before the most recent pregnancy (aOR 1.3, 95% confidence interval (CI): 0.9–1.8). Predictors of PPD among HIV positive women were living with friends/in a house-share (aOR 0.5 for house-share vs. own home, 95% CI: 0.3–0.9), and attending antenatal care (ANC) for the most recent pregnancy (aOR 0.2 for ANC attendance vs. no ANC attendance, 95% CI: 0.0–0.5). Living with friends/in a house-share was also a predictor of PPD among HIV negative women (aOR 0.4 for house-share vs. own home, 95% CI: 0.2–0.8).

**Conclusions and recommendations:**

Targeted symptom screening based on identified risk factors should be considered for postpartum women to increase PPD case-finding and referral to specialised social support services.

## Background

Globally, maternal postpartum depression (PPD) is a major risk factor for non-obstetric postnatal maternal and child morbidity as well as mortality [1–3]. The proportion of postnatal women diagnosed with PPD varies widely from 10–15% in high-income countries to 30–60% in sub-Saharan Africa [4–7]. Untreated PPD can lead to chronic depression, disruptions of family and marital relationships, and can cause long-term health and developmental problems in children of affected women [5, 8]. Early diagnosis and treatment of PPD improves the prognosis for both mother and child [9, 10]. However, in high-income countries, almost 50% of PPD cases are undiagnosed, with only 30% of diagnosed cases receiving treatment [11–13]. In South Africa, an estimated 30–50% of women are likely to develop PPD [7, 14, 15], and although PPD-specific treatment estimates are unavailable, overall 75% of patients’ mental health problem, including depression, do not access treatment in South Africa [16, 17].

Infants are affected by their mother’s mental health problems as they depend on their mothers for their developmental and nutritional needs [18, 19]. Women with depressive symptoms report lower rates of breastfeeding compared to women without depressive symptoms (49% vs 61%) [19, 20]. A meta-analysis of studies from developing countries found that children of mothers with PPD were 50% more likely to be underweight or stunted [18]. Furthermore, depressed mothers are often disengaged from their infants which may lead to slower cognitive development, behavioural problems and long-term psychological difficulties [19, 21–24]. The quality of infant-mother relationships appears to predict behavioural problems and disruption of cognitive abilities in children [23]. Evidence also suggests that children of depressed mothers also experience higher mortality rates. A study conducted in Taiwan, which examined mortality of children up to age five years, found that children were at a 47% greater risk of death if their mother was depressed [25]. Similarly, a cohort study in Ghana reported a nearly three-fold increase in the risk of mortality by six months among infants born to women diagnosed with PPD [3].

Predictors of PPD in the South African context include relational factors such as marital status and partner’s financial/ moral support, an unplanned/unwelcome baby, infant health conditions as well as the mother’s educational attainment and employment situation [7, 14]. Additionally, personal or family history of depression, recent stressful life events, high childcare stress, low self-esteem and neuroticism are important factors [24, 26, 27]. However, the impact of exposure to HIV care either through the HIV diagnostic procedures and ARV treatment before pregnancy in mitigating the risk of PPD among HIV positive women is not well described.

In general, rates of PPD are high among women living with HIV, with PPD rates of above 40% in high HIV prevalence settings [5, 7, 15, 28, 29]. Many women learn about their HIV infection during, or shortly after a pregnancy which can adversely impact on their mental health and compromise their participation in antenatal care (ANC) including the prevention of mother-to-child transmission (PMTCT) programs and antiretroviral therapy adherence [6, 30, 31]. Therefore, screening, referral for diagnosis and treatment of PPD among postpartum HIV-1 infected women is vital for the attainment of the HIV care and treatment goals for both the mothers and their infants [32, 33].

It is unclear whether psychosocial support associated with HIV care/treatment positively impacts on the risk of PPD among HIV-1 infected with pre-pregnancy HIV care experience. In this study, we set out to measure the prevalence of PPD comparing HIV-1 infected women with pre-pregnancy HIV care experience, newly diagnosed (in latest pregnancy) HIV-1 infected women and HIV negative women, and to identify predictors of having depressive symptoms among these women.

## Materials and methods

### Study design and population

**Fig 1:**
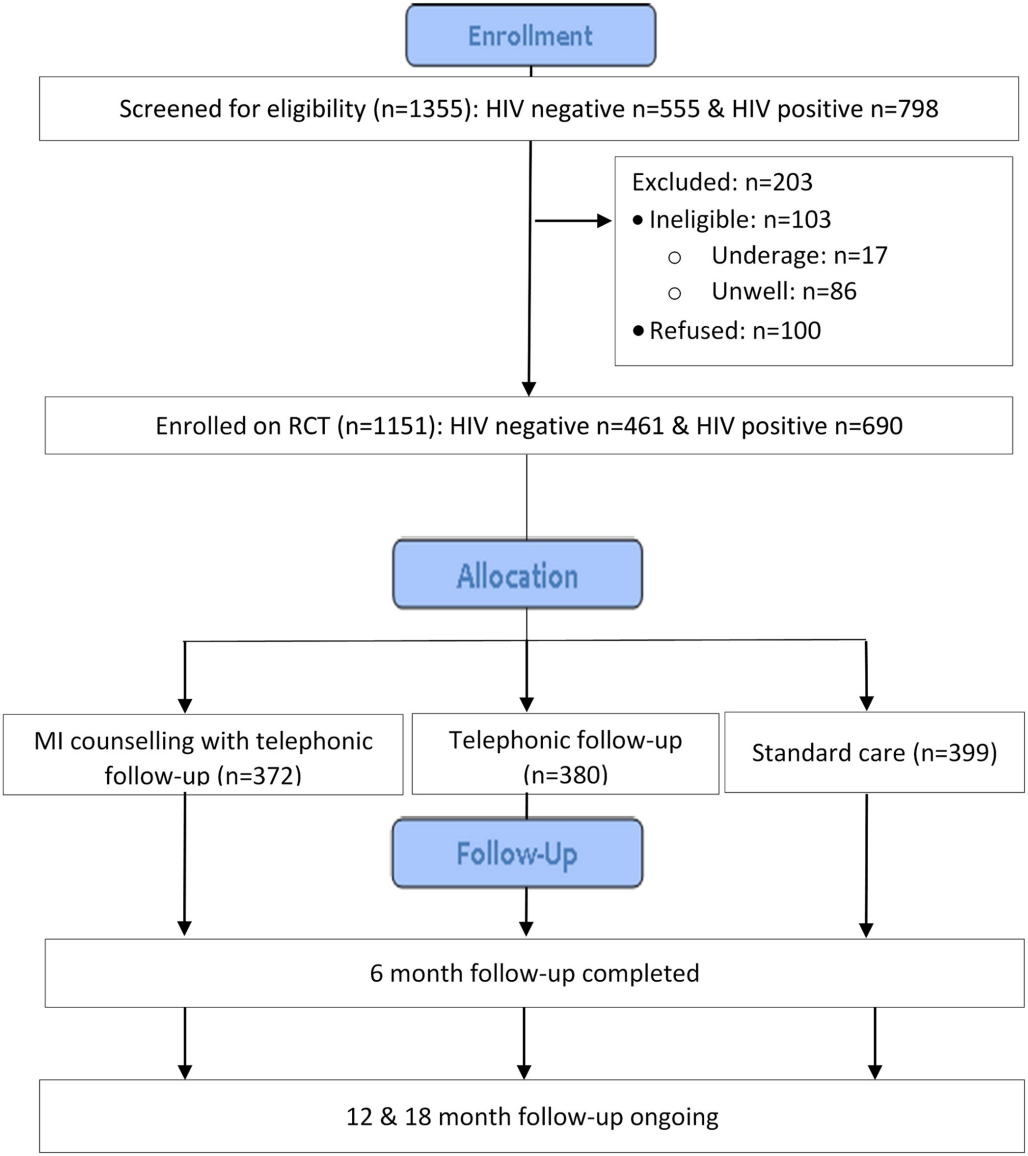
CONSORT diagram of participant enrolment and follow-up in the parent study (RCT).

This data was collected as part of the baseline survey of a randomised controlled trial (RCT) (Pan African Clinical Trials Registry: PACTR201809886446171) among adult (≥18 years) women who gave birth up to 30 days before the date of enrolment at Midwife Obstetric Units (MOUs) based in Tshwane, Ekurhuleni and Johannesburg Metropolitan districts in the Gauteng Province, South Africa. New mothers were recruited consecutively via referrals from nurses at participating facilities and interviewed on the day of their first post-natal visit (scheduled three to six days after delivery). Study staff screened potential eligible women using study eligibility criteria, provided more information regarding the study, and obtained written informed consent using informed consent translated from English into Sotho and Zulu and administered in the participant’s preferred language (English, Sotho or Zulu). Study enrolment was conducted from October 2016 to January 2018. The sampling for the RCT was stratified by HIV status, and participants were randomised to either Active Tracing, Active Tracing with Motivational Interviewing (MI) counselling support tracing approaches or standard of care. Six month follow-up for the RCT has been completed, and the 12 and 18 month follow-up is ongoing (Fig 1). This analysis includes cross-sectional data collected using baseline questionnaire at study enrolment, which included 690 HIV-1 infected and 461 HIV-1 uninfected women (Fig 1). Participant demographic, socio-economic and contextual data were collected using an interviewer-administered structured questionnaire translated in English, Sotho and Zulu and administered in the participant’s preferred language (English, Sotho or Zulu).

### Analytical variables

PPD was measured using the CES-D 10 scale, a 10-question four-point scale (scores range 0 to 3) that measures general depressive symptoms experienced up to 7 days before the interview date [34–36]. The total score ranged from 0 to 30 with higher total scores reflecting greater frequency of depression (Cronbach’s alpha = 0.83). We created a variable for PPD categorised into no depression (CES-D 10 total score <5), low to medium depression (CES-D 10 total score ≥5 and <10) and major depressive symptoms (CES-D 10 total score≥10) [37, 38]. The CES-D 10 scale has been validated in South Africa and other low and middle-income countries (LMIC) and is used as a screening tool for PPD [35, 39–41].

Additional variables assessed included socio-demographic factors including age, highest education completed, marital status, employment status and work times (all-day, shift-work) and income-source. Perceived social support (PSS) was measured using a six-item scale in which participants indicated their overall level of satisfaction with available support given in each area [34]. Rating of overall satisfaction score for each item ranged from one to five. Total scores were categorised as either "high PSS” (score ≥26) or “medium PSS” (score ≤25) based on the distribution of scores in the sample. HIV knowledge was measured among HIV-positive women based on responses to 12 HIV knowledge questions, each with a possible score of 0 and 1 for incorrect and correct response respectively. Total knowledge scores were categorised as “Low” (score<11) or “Medium to high” (score ≥11) HIV knowledge. We also assessed factors related to ANC attendance, location and type of housing, whether the latest pregnancy was planned, new-born baby’s gender, number of child dependents (own and others'), support during pregnancy, and expected childcare support postpartum. Ethics approval for this study was obtained from the Wits Human Research Ethics Committee (Medical) (HREC No.M151041). All personal identifiers were removed from the final analytic data.

### Statistical analysis

Descriptive analysis was used to summarise participant characteristics at study enrolment. Categorical variables were tabulated using frequencies and percentages. Continuous variables were described using medians and interquartile ranges (IQR) where appropriate. We used Ordinal logistic regression to identify predictors of postpartum depression and the associated 95% confidence intervals (95% CI). As we could only identify prediction and not causation, factors identified with a univariate p-value <0.1 and priori variables of importance and predictors were included in the multivariate model. Significance level was set at 0.05 for the multivariate model. The Likelihood ratio test was used to test for adherence to the proportional odds assumption. We report adjusted odds ratio (aOR) and 95% confidence intervals (CIs). Data analysis was conducted using STATA version 14 (StataCorp, College Station, TX).

## Results

### Socio-demographic factors

Table 1 shows demographic and contextual characteristics of study participants which included 1151 postpartum women of a median age of 29 years (IQR: 25–33) at study enrolment, of which over 80% had at least some secondary school education. A total of 461 (40.1%) were HIV negative, and 690 (59.9%) were HIV infected. Just over half (364/690 or 52.7%) of the HIV infected women were diagnosed with HIV before their latest pregnancy, and among these 152/364 (41.8%) had a prior (in-pregnancy) HIV diagnosis and had prior experience of the PMTCT program. A total of 249 (21.6%) women were married, with a higher marriage percentage among HIV negative mothers (26.9%) compared to HIV positive mothers (125/690 or 18.1%). Overall, 78.4% of women were in non-marital relationships, with 44.6% cohabiting with a partner. Over two-thirds of the women lived with a partner or spouse (64.6%), and 46.5% lived in their own home. Nearly two thirds (63.3%) of the mothers were unemployed, with 19.5% not seeking employment. Unemployment rates were similar across HIV status. However, unemployed HIV-infected infected women (47.0%) were more likely to be searching for work compared to HIV negative women (27.5%). The majority of women reported their spouse/ partner to be their primary source of income (56.2%), with a higher proportion of HIV negative mothers relying on spousal/ partner support than their HIV-infected counterparts (61.0% vs 53.2%). Over half of the women, 674 (58.6%), had high perceived social support.

**Table 1:** Participant characteristics (all women).

	HIV negative	Diagnosed during latest pregnancy	Pre-pregnancy HIV diagnosis	Total
	461 (40.1) No. (%)	326 (28.3) No. (%)	364 (31.6) No. (%)	1151 (100.0) No. (%)
**Age (years)**				
18–25	182 (39.5)	96 (29.4)	53 (14.6)	331 (28.8)
26–30	147 (31.9)	105 (32.2)	90 (24.7)	342 (29.7)
31–35	92 (20.0)	81 (24.8)	122 (33.5)	295 (25.6)
>35	40 (8.7)	44 (13.5)	99 (27.2)	183 (15.9)
**Highest level of education**				
Tertiary level	61 (13.2)	50 (15.3)	38 (10.4)	149 (12.9)
Matric	146 (31.7)	86 (26.4)	73 (20.1)	305 (26.5)
High school	234 (50.8)	180 (55.2)	229 (62.9)	643 (55.9)
Primary school or less	20 (4.3)	10 (3.1)	23 (6.3)	53 (4.6)
Missing	-	-	1 (0.3)	1 (0.1)
**Marital status**				
Married	124 (26.9)	51 (15.6)	74 (20.3)	249 (21.6)
In a relationship (living together)	178 (38.6)	155 (47.5)	179 (49.2)	512 (44.5)
In a relationship (not living together)	139 (30.2)	103 (31.6)	95 (26.1)	337 (29.3)
Not in a relationship	19 (4.1)	16 (4.9)	15 (4.1)	50 (4.3)
Missing	1 (0.2)	1 (0.3)	1 (0.3)	3 (0.3)
**Accommodation**				
Own home	173 (37.5)	102 (31.3)	144 (39.6)	419 (36.4)
Family’s home	153 (33.2)	118 (36.2)	113 (31.0)	384 (33.4)
Friends or house-share	134 (29.1)	105 (32.2)	107 (29.4)	346 (30.1)
Missing	1 (0.2)	1 (0.3)	-	2 (0.2)
**Participant lives with**				
With partner/spouse	293 (63.6)	202 (62.0)	249 (68.4)	744 (64.6)
Parents/relatives	147 (31.9)	101 (31.0)	78 (21.4)	326 (28.3)
Alone/with children	19 (4.1)	19 (5.8)	32 (8.8)	70 (6.1)
Missing	2 (0.4)	4 (1.2)	5 (1.4)	11 (1.0)
**Location of primary house (when living in secondary house)**				
current house	168 (36.4)	135 (41.4)	153 (42)	456 (39.6)
same province	26 (5.6)	24 (7.4)	26 (7.1)	76 (6.6)
Another province/rural-area	120 (26.0)	95 (29.1)	112 (30.8)	327 (28.4)
Another country	147 (31.9)	71 (21.8)	72 (19.8)	290 (25.2)
Missing	-	1 (0.3)	1 (0.3)	2 (0.2)
**Accommodation type**				
House/Flat/Brick structure	139 (30.2)	106 (32.5)	122 (33.5)	367 (31.9)
House/room/flat in backyard	220 (47.7)	160 (49.1)	172 (47.3)	552 (48.0)
Informal dwelling/shack	101 (21.9)	60 (18.4)	70 (19.2)	231 (20.1)
Missing	1 (0.2)	-	-	1 (0.1)
**Employment status**				
Employed-work all day	113 (24.5)	95 (29.1)	97 (26.6)	305 (26.5)
Employed-shift work	41 (8.9)	36 (11.0)	40 (11.0)	117 (10.2)
Unemployed (not job hunting)	127 (27.5)	48 (14.7)	50 (13.7)	225 (19.5)
Unemployed (job hunting)	180 (39.0)	147 (45.1)	177 (48.6)	504 (43.8)
**Primary source of income/ finances**				
Paid job, salary or business	108 (23.4)	107 (32.8)	101 (27.7)	316 (27.5)
Government grant	5 (1.1)	20 (6.1)	35 (9.6)	60 (5.2)
Spouse/ partner	280 (60.7)	161 (49.4)	206 (56.6)	647 (56.2)
Parents/ relatives	66 (14.3)	36 (11.0)	21 (5.8)	123 (10.7)
Missing	2 (0.4)	2 (0.6)	1 (0.3)	5 (0.4)
**Sex of new-born baby**				
Male	225 (48.8)	169 (51.8)	181 (49.7)	575 (50.0)
Female	236 (51.2)	157 (48.2)	183 (50.3)	576 (50.0)
**Number of other children of any age**				
0 children	138 (29.9)	60 (18.4)	30 (8.2)	228 (19.8)
=>1 children	306 (66.4)	259 (79.4)	329 (90.4)	894 (77.7)
Missing	17 (3.7)	7 (2.1)	5 (1.4)	29 (2.5)
**Latest pregnancy planned?**				
No	209 (45.3)	182 (55.8)	195 (53.6)	586 (50.9)
Yes	252 (54.7)	144 (44.2)	169 (46.4)	565 (49.1)
**Baby father’s involvement in the pregnancy?**				
Involved	439 (95.2)	305 (93.6)	341 (93.7)	1,085.0 (94.3)
Not involved	22 (4.8)	21 (6.4)	23 (6.3)	66 (5.7)
**Perceived greatest supporter during the latest pregnancy**				
Partner	247 (53.6)	189 (58.0)	190 (52.2)	626 (54.4)
Baby father(if not partner)	99 (21.5)	71 (21.8)	98 (26.9)	268 (23.3)
Family/friends/Other	112 (24.3)	65 (19.9)	74 (20.3)	251 (21.8)
Missing	3 (0.7)	1 (0.3)	2 (0.5)	6 (0.5)
**Expected main childcare supporter**				
Partner	187 (40.6)	142 (43.6)	146 (40.1)	475 (41.3)
Baby father(if not partner)	88 (19.1)	65 (19.9)	97 (26.6)	250 (21.7)
Family/friends/Other	184 (39.9)	119 (36.5)	121 (33.2)	424 (36.8)
Missing	2 (0.4)	-	-	2 (0.2)
**ANC attendance**				
No	5 (1.1)	4 (1.2)	6 (1.6)	15 (1.3)
Yes	454 (98.5)	322 (98.8)	358 (98.4)	1,134.0 (98.5)
Missing	2 (0.4)	-	-	2 (0.2)
**HIV knowledge**				
Low	-	96 (29.4)	115 (31.6)	211 (30.6)
Medium to high	-	227 (69.6)	247 (67.9)	474 (68.7)
Missing	-	3 (0.9)	2 (0.6)	5 (0.7)
**Perceived social support (PSS)**				
High PSS	298 (64.6)	172 (52.8)	204 (56.0)	674 (58.6)
Medium PSS	163 (35.4)	154 (47.2)	160 (44.0)	477 (41.4)
**Post-partum depression (PPD)**				
No depression	332 (72.0)	257 (78.8)	274 (75.3)	863 (75.0)
Low to medium depression	79 (17.1)	43 (13.2)	46 (12.6)	168 (14.6)
Major depressive symptoms	50 (10.9)	26 (8.0)	44 (12.1)	120 (10.4)

ANC: Antenatal care; HIV: human immunodeficiency virus; ART: antiretroviral treatment

### Pregnancy history and social support

Only 19.8% of women were primiparous and for half of them (50.9%), the latest pregnancy was unplanned. The partner/baby’s father support during the latest pregnancy was generally high (75.5% HIV negative vs 79.6% of HIV-infected mothers). However, the expected partner/father support in childcare activities was higher (65.2% vs. 60.0% among HIV negative women) for HIV-infected women with lower expected family support for childcare (34.8%) compared to 39.9% among HIV negative women. Overall, ANC attendance was high with 98.5% having any ANC attendance during the latest the pregnancy. The majority of HIV-infected women had medium to high HIV knowledge (69.2%), 70% among women with pre-pregnancy diagnosis and 68% among newly diagnosed women. Perceived social support was higher among HIV negative women (64.6%) compared to HIV-infected women (54.4%).

### Prevalence of PPD

Overall, 168/1151 (14.6%) of the sample had low to medium PPD and 120/1151 (10.4%) women screened positive for major PPD, 10.9% among HIV negative women and 10.1% among HIV positive women. Among HIV positive women, a higher proportion (12.1%) of those with a pre-pregnancy HIV diagnosis screened positive for major PPD compared to women with in-pregnancy HIV diagnosis (8.0%).

### Predictors of PPD (multivariable analysis)

Table 2 shows crude and adjusted estimates from the ordinal logistic regression model with 95% CIs for experiencing low to medium PPD and major depression respectively.

**Table 2:** Predictors of postpartum depression by HIV status.

	PPD		HIV positive women (N = 461)		HIV negative women (N = 690)	
	Low to medium depression *168 (14*.*6)*	Major Depression *120 (10*.*4*)	Crude	Adjusted	Crude	Adjusted
	*n (%)*	*n (%)*	*OR (95% CI)*	*aOR (95% CI)*	*OR (95% CI)*	*aOR (95% CI)*
**HIV status**						
HIV negative	79 (17.1)	50 (10.8)				
HIV positive	89 (25.8)	70 (20.1)				
**Timing of HIV diagnosis**						
HIV negative	79 (17.1)	50 (10.8)				
Diagnosed during latest pregnancy	43 (13.2)	26 (8)	1			
Pre-pregnancy diagnosis	46 (12.6)	44 (12.1)	1.3 (0.9–1.8)			
**Age (years)**						
18–25	71 (21.5)	28 (8.5)	1		1	1
26–30	41 (12)	36 (10.5)	0.7 (0.5–1.2)		0.8 (0.5–1.2)	0.8 (0.5–1.3)
31–35	36 (12.2)	40 (13.6)	0.8 (0.5–1.3)		1.2 (0.7–2.0)	1.2 (0.7–2.1)
>35	20 (10.9)	16 (8.7)	0.7 (0.4–1.2)		0.5 (0.2–1.1)	0.5 (0.2–1.3)
**Highest level of education**						
Tertiary level	21 (14.1)	16 (10.7)	1		1	
Matric	52 (17)	29 (9.5)	0.8 (0.4–1.5)		1.5 (0.7–2.9)	
High school	90 (14)	65 (10.1)	0.8 (0.5–1.4)		1.2 (0.6–2.3)	
Primary school or less	5 (9.4)	9 (17)	0.7 (0.3–1.9)		2.3 (0.8–6.6)	
**Marital status**						
Married	29 (11.6)	24 (9.6)	1		1	1
In a relationship (living together)	71 (13.9)	52 (10.2)	1.1 (0.7–1.8)		1.3 (0.8–2.2)	1.2 (0.7–2.1)
In a relationship (not living together)	60 (17.8)	34 (10.1)	1.4 (0.8–2.4)		1.4 (0.8–2.4)	1.1 (0.6–2.1)
Not in a relationship	8 (16)	10 (20)	1.6 (0.7–3.9)		3.6 (1.4–9.7)	2.3 (0.8–6.6)
**Accommodation**						
Own home	56 (13.4)	58 (13.8)	1	1	1	1
Family’s home	69 (18)	41 (10.7)	1.1 (0.7–1.6)	1.0 (0.5–1.7)	0.9 (0.6–1.5)	0.7 (0.5–1.2)
Friends or house-share	42 (12.1)	21 (6.1)	0.5 (0.3–0.8)	0.5 (0.3–0.9)	0.7 (0.4–1.1)	0.4 (0.2–0.8)
**Participant lives with**						
With partner/spouse	98 (13.2)	73 (9.8)	1	1	1	
Parents/relatives	61 (18.7)	38 (11.7)	1.6 (1.1–2.3)	1.2 (0.7–2.2)	1.2 (0.8–1.9)	
Alone/with children	9 (12.9)	7 (10)	1.0 (0.5–2.1)	1.1 (0.5–2.4)	1.0 (0.4–2.8)	
**Location of primary house (when living in secondary house)**						
current house	74 (16.2)	52 (11.4)	1	1	1	
same province	10 (13.2)	4 (5.3)	0.6 (0.3–1.3)	0.9 (0.4–2.1)	0.6 (0.2–1.5)	
Another province/rural-area	38 (11.6)	35 (10.7)	0.7 (0.4–1.0)	0.9 (0.5–1.5)	1.0 (0.6–1.6)	
Another country	46 (15.9)	29 (10)	1.0 (0.6–1.5)	1.4 (0.8–2.6)	0.8 (0.5–1.3)	
**Accommodation type**						
House/Flat/Brick structure	67 (18.3)	40 (10.9)	1		1	
House/room/flat in backyard	78 (14.1)	61 (11.1)	0.9 (0.6–1.3)		0.8 (0.5–1.2)	
Informal dwelling/shack	23 (10)	19 (8.2)	0.7 (0.4–1.2)		0.4 (0.2–0.7)	
**Employment status**						
Employed-work all day	39 (12.8)	26 (8.5)	1	1	1	
Employed-shift work	14 (12)	14 (12)	1.5 (0.8–2.9)	1.9 (0.9–3.6)	0.8 (0.3–1.8)	
Unemployed (not job hunting)	38 (16.9)	22 (9.8)	1.1 (0.6–2.1)	0.7 (0.3–1.5)	1.3 (0.7–2.2)	
Unemployed (job hunting)	77 (15.3)	58 (11.5)	1.6 (1.0–2.5)	1.0 (0.5–2.0)	1.1 (0.6–1.8)	
**Primary source of income/ finances**						
Paid job, salary or business	38 (12)	26 (8.2)	1	1	1	
Government grant	5 (8.3)	10 (16.7)	1.5 (0.7–3.1)	1.7 (0.7–4.1)	2.2 (0.4–13.1)	
Spouse/ partner	102 (15.8)	66 (10.2)	1.4 (0.9–2.2)	1.8 (0.9–3.5)	1.2 (0.7–2.0)	
Parents/ relatives	22 (17.9)	18 (14.6)	2.1 (1.1–4.1)	1.9 (0.7–4.7)	1.5 (0.8–3.0)	
**Sex of new-born baby**						
Male	83 (14.4)	57 (9.9)	1	1	1	
Female	85 (14.8)	63 (10.9)	1.4 (1.0–1.9)	1.4 (0.9–2.0)	0.8 (0.5–1.2)	
**Number of other children of any age**						
0 children	43 (18.9)	21 (9.2)	1		1	
=>1 children	120 (13.4)	99 (11.1)	0.9 (0.5–1.5)		0.9 (0.6–1.5)	
**Latest pregnancy planned?**						
No	91 (15.5)	69 (11.8)	1	1	1	
Yes	77 (13.6)	51 (9)	0.7 (0.5–0.9)	0.7 (0.5–1.0)	0.9 (0.6–1.4)	
**Baby father’s involvement in the pregnancy?**						
Involved	160 (14.7)	110 (10.1)	1		1	
Not involved	8 (12.1)	10 (15.2)	1.0 (0.5–2.1)		1.7 (0.7–4.1)	
**Perceived greatest supporter during the latest pregnancy**						
Partner	88 (14.1)	56 (8.9)	1		1	
Baby father(if not partner)	39 (14.6)	34 (12.7)	1.3 (0.8–1.9)		1.3 (0.8–2.2)	
Family/friends/Other	39 (15.5)	29 (11.6)	1.4 (0.9–2.2)		1.1 (0.7–1.8)	
**Expected main childcare supporter**						
Partner	61 (12.8)	41 (8.6)	1		1	1
Baby father(if not partner)	36 (14.4)	29 (11.6)	1.1 (0.7–1.8)		1.7 (1.0–2.9)	1.6 (0.9–2.8)
Family/friends/Other	71 (16.7)	50 (11.8)	1.4 (0.9–2.0)		1.5 (1.0–2.4)	1.3 (0.8–2.3)
**ANC attendance**						
No	5 (33.3)	5 (33.3)	1	1	1	
Yes	163 (14.4)	115 (10.1)	0.1 (0.0–0.4)	0.2 (0.0–0.5)	0.3 (0.1–1.5)	
**HIV knowledge**						
Low	28 (13.3)	24 (11.4)	1			
Medium to high	61 (12.9)	45 (9.5)	0.9 (0.6–1.3)			
**Perceived social support (PSS)**						
High PSS	97 (14.4)	59 (8.8)	1		1	1
Medium PSS	71 (14.9)	61 (12.8)	1.3 (0.9–1.8)		1.4 (1.0–2.2)	1.3 (0.8–2.0)

OR: Odds ratio; aOR: Adjusted odds ratio; HIV: human immunodeficiency virus; ANC: Antenatal care

#### HIV-infected

Among HIV-infected women, there was no difference in odds of experiencing PPD by timing of HIV diagnosis in the univariate analysis (OR 1.3, 95%CI: 0.9–1.8). Living with friends or house-mates was associated with lower risk of experiencing PPD (aOR 0.5, 95%CI: 0.3–0.9), as well as having attended antenatal care during the latest pregnancy (aOR 0.2, 95% CI: 0.0–0.5).

#### HIV negative

Among HIV negative women living with friends or house-mates was associated with lower risk of experiencing PPD (aOR 0.4, 95% CI: 0.2–0.8).

On further analysis in a multivariable analysis including all women, newly diagnosed HIV-infected women were less likely to be depressed compared to HIV negative women (aOR 0.7, 95% CI: 0.5–0.99), while there was no difference in odds of experiencing depression between HIV negative women and those with pre-pregnancy HIV diagnosis.

## Discussion

This is one of the largest studies assessing PPD in the sub-Sahara Africa setting, and one of the few that looks at differences in PPD-based HIV status and timing of HIV diagnosis. Results from our study show that a quarter of women had depressive tendencies after delivery, but only 10.9% of HIV negative and 10.1% of HIV positive women showed signs of major PPD.

The risk of major PPD in both our HIV positive and negative women is lower than previously reported PPD prevalence in low and middle-income countries, including South Africa, and is much closer to rates found in high-income countries [4–6]. Variations in PPD rates possibly emanate from screening tool preferences as well as the varying definitions for PPD across studies, with very few elaborating on the severity of the reported risk of PPD. A large prospective cohort study in the general postpartum population in Soweto, Johannesburg, reported PPD rates of 16.4% using a total score threshold of 20 on the Pitt Depression Questionnaire depressive symptoms [42]. Previous smaller studies in sub-Saharan Africa, which defined major PPD at total score thresholds ranging from 11–15 on the Edinburg Postnatal Depression Scale-10 (EPDS-10) [43], reported PPD prevalence of 33% in a mixed Zimbabwean cohort HIV-1 infected cohort [28], 45.1% was found among HIV-infected women in rural Mpumalanga (South Africa) [44].

However, our study consecutively enrolled postnatal women who delivered both at low-risk (MOU) and hospital facilities and the setting of enrolment (MOU) could have systematically excluded women with high-risk pregnancies who may also have greater risk of major PPD. There is also a possibility that symptoms were played down/ underreported due to the stigma associated with mental health disorders in many African cultures [45, 46]. Women may worry that their child caring capacity may be called into question and they may also be reluctant to take up treatment interventions involving prescription drugs while breastfeeding [10, 17, 47]. On average 50% of depressed women often go undiagnosed, with only 20% of those diagnosed seek treatment [11–13, 47].

We hypothesised that HIV-infected women with pre-pregnancy HIV diagnosis and hopefully some prior experience of HIV care would be more resilient and be at lower risk of major PPD than newly diagnosed HIV-infected women. However, we found no difference in major PPD by the timing of HIV diagnosis among HIV infected women, but found that women diagnosed with HIV during their latest pregnancy were less likely to experience PPD than HIV negative women. These results are contrary to results from two previous studies in Zambia and South Africa that found an increased risk of PPD among women who discovered their HIV-1 diagnosis during the last pregnancy [44, 48]. Similar to previous studies, we found no difference in PPD by HIV status [28, 33]. The impact of the PMTCT program and increased life expectancy of HIV-infected individuals may have lessened women’s concerns about the risk of HIV transmission to their infants as well as fears of premature death [49–51]. Addressing depression in HIV negative women remains a crucial HIV-preventive measure as untreated depression is associated with negative coping behaviours (unprotected sex, having multiple and concurrent sexual partners, use of illicit substances) that increase the risk for HIV [52].

Predictors of PPD identified among the combined sample of HIV-infected and negative mothers include living with friends or sharing a house with others, antenatal care attendance and timing of HIV diagnosis when compared to HIV negative women. In many settings including South Africa, older female family members experienced in child care are customarily tasked with providing childcare support and guidance to new mothers. Depending on the context, these family members may come and stay with the new mother in her own home, or the new mother may move to her family home to access this support. Women who live with friends or sharing with others may, therefore, have lower expectations of this type of support. Antenatal care engagement may expose women to services that may mitigate some of the pregnancy, labour and childcare related stressors that may contribute to the risk of PPD.

Perceived social support has been previously reported as a critical factor in mitigation of PPD [5, 28, 53, 54], but it wasn’t shown as important in our cohort of women which may need to be explored further to understand what factors could be helping them to cope positively with perinatal related stressors, as well as HIV among our HIV infected women.

The cross-sectional study design limits the interpretation of the study results, and causal associations cannot be inferred. Depressive symptoms were self-reported using a validated tool, but participant recall and social desirability bias cannot be excluded. Although the CES-D 10 scale is a screening tool and not a diagnostic interview, it has been shown to have high levels of sensitivity and specificity for postpartum depression [36]. The study was conducted in an urban setting with participants hailing from a mixture of formal and informal settlements, and the study results may not be generalizable to rural settings. Child outcomes were not measured which would have strengthened the results.

## Conclusions

Our results show a lower prevalence of PPD than previously reported, with no difference noted by HIV status, possibly indicating increased normalisation of HIV disease among urban populations in South Africa. However, efforts to identify depressed mothers using targeted symptom screening based on risk factors, and linked to effective treatment interventions remain essential in improving postpartum mother and child outcomes.

## References

[1] Stocky A, Lynch J. Acute psychiatric disturbance in pregnancy and the puerperium. Best Practice & Research Clinical Obstetrics & Gynaecology. 2000;14(1):73–87. 10.1053/beog.1999.0064 10789261

[2] World Health Organization. Depression: A Global Crisis. World Mental Health Day, October 10 2012. World Federation for Mental Health, Occoquan, Va, USA. 2012.

[3] Weobong B, ten Asbroek AH, Soremekun S, Gram L, Amenga-Etego S, Danso S, et al Association between probable postnatal depression and increased infant mortality and morbidity: findings from the DON population-based cohort study in rural Ghana. BMJ open. 2015;5(8):e006509 10.1136/bmjopen-2014-006509 26316646 PMC4554911

[4] Madlala S, Kassier S. Antenatal and postpartum depression: effects on infant and young child health and feeding practices. South African Journal of Clinical Nutrition. 2017:1–7.

[5] Peltzer K, Shikwane M. Prevalence of postnatal depression and associated factors among HIV-positive women in primary care in Nkangala district, South Africa. Southern African Journal of HIV Medicine. 2011;12(4):24–8.

[6] Antelman G, Kaaya S, Wei R, Mbwambo J, Msamanga GI, Fawzi WW, et al Depressive symptoms increase risk of HIV disease progression and mortality among women in Tanzania. JAIDS Journal of Acquired Immune Deficiency Syndromes. 2007;44(4):470–7. 10.1097/QAI.0b013e31802f1318 17179766 PMC6276368

[7] Mokwena K, Shiba D. Prevalence of postnatal depression symptoms in a primary health care clinic in Pretoria, South Africa: management of health care services. African Journal for Physical Health Education, Recreation and Dance. 2014;20(Supplement 1):116–27.

[8] Castle J. Early detection of postpartum depression: Screening in the first two to three days. J Lancaster Gen Hosp Winter. 2008;2009(3):4.

[9] Dennis CL, Dowswell T. Psychosocial and psychological interventions for preventing postpartum depression. The Cochrane Library. 2013. 10.1002/14651858.CD001134.pub3 PMC11936315 23450532

[10] National Collaborating Centre for Mental Health, editor Antenatal and Postnatal Mental Health: Clinical Management and Service Guidance: Updated edition2014: British Psychological Society. 26180865

[11] Appleby L, Hirst E, Marshall S, Keeling F, Brind J, Butterworth T, et al The treatment of postnatal depression by health visitors: impact of brief training on skills and clinical practice. Journal of Affective Disorders. 2003;77(3):261–6. 14612226 10.1016/s0165-0327(02)00145-3

[12] Benvenuti P, Valoriani V, Vanni D. Prevention of postnatal depression. Clinical Neuropsychiatry. 2006;3(1):39–56.

[13] England S, Ballard C, George S. Chronicity in postnatal depression. The European journal of psychiatry. 1994;8(2):93–6.

[14] Stellenberg EL, Abrahams JM. Prevalence of and factors influencing postnatal depression in a rural community in South Africa. African journal of primary health care & family medicine. 2015;7(1):1–8. 10.4102/phcfm.v7i1.874 PMC4729123 26842515

[15] Rochat TJ, Tomlinson M, Newell M-L, Stein A. Detection of antenatal depression in rural HIV-affected populations with short and ultrashort versions of the Edinburgh Postnatal Depression Scale (EPDS). Archives of women’s mental health. 2013;16(5):401–10. 10.1007/s00737-013-0353-z 23615932 PMC3778840

[16] Seedat S, Stein D, Herman A, Kessler R, Sonnega J, Heeringa S, et al Twelve-month treatment of psychiatric disorders in the South African Stress and Health study (World Mental Health survey initiative). Social psychiatry and psychiatric epidemiology. 2008;43(11):889–97. 10.1007/s00127-008-0399-9 18677573 PMC3222914

[17] Kathree T, Selohilwe OM, Bhana A, Petersen I. Perceptions of postnatal depression and health care needs in a South African sample: the “mental” in maternal health care. BMC women’s health. 2014;14(1):140. 25389015 10.1186/s12905-014-0140-7 PMC4231193

[18] Surkan PJ, Kennedy CE, Hurley KM, Black MM. Maternal depression and early childhood growth in developing countries: systematic review and meta-analysis. Bulletin of the World Health Organization. 2011;89(8):607–15. 10.2471/BLT.11.088187 PMC3150769 21836759

[19] Wachs TD, Black MM, Engle PL. Maternal depression: a global threat to children’s health, development, and behavior and to human rights. Child Development Perspectives. 2009;3(1):51–9.

[20] Woolhouse H, James J, Gartland D, McDonald E, Brown SJ. Maternal depressive symptoms at three months postpartum and breastfeeding rates at six months postpartum: Implications for primary care in a prospective cohort study of primiparous women in Australia. Women and Birth. 2016;29(4):381–7. 10.1016/j.wombi.2016.05.008 27450375

[21] Field T, Hernandez-Reif M, Diego M, Feijo L, Vera Y, Gil K, et al Still-face and separation effects on depressed mother-infant interactions. Infant Mental Health Journal. 2007;28(3):314–23. 10.1002/imhj.20138 28640469

[22] Weinberg MK, Tronick EZ, Beeghly M, Olson KL, Kernan H, Riley JM. Subsyndromal depressive symptoms and major depression in postpartum women. American Journal of Orthopsychiatry. 2001;71(1):87 11271721 10.1037/0002-9432.71.1.87

[23] Paris R, Bolton RE, Weinberg MK. Postpartum depression, suicidality, and mother-infant interactions. Archives of women’s mental health. 2009;12(5):309 10.1007/s00737-009-0105-2 19728036

[24] Robertson E, Grace S, Wallington T, Stewart DE. Antenatal risk factors for postpartum depression: a synthesis of recent literature. General hospital psychiatry. 2004;26(4):289–95. 10.1016/j.genhosppsych.2004.02.006 15234824

[25] Chen Y, Tsai S, Lin H. Increased mortality risk among offspring of mothers with postnatal depression: a nationwide population-based study in Taiwan. Psychological medicine. 2011;41(11):2287–96. 10.1017/S0033291711000584 21524332

[26] Stewart DE, Robertson E, Dennis C-L, Grace SL, Wallington T. Postpartum depression: Literature review of risk factors and interventions. Toronto: University Health Network Women’s Health Program for Toronto Public Health 2003.

[27] Verreault N, Da Costa D, Marchand A, Ireland K, Dritsa M, Khalifé S. Rates and risk factors associated with depressive symptoms during pregnancy and with postpartum onset. Journal of psychosomatic obstetrics & gynecology. 2014;35(3):84–91. 25123985 10.3109/0167482X.2014.947953

[28] Chibanda D, Mangezi W, Tshimanga M, Woelk G, Rusakaniko S, Stranix-Chibanda L, et al Postnatal depression by HIV status among women in Zimbabwe. Journal of Women’s Health. 2010;19(11):2071–7. 10.1089/jwh.2010.2012 20849286

[29] Yator O, Mathai M, Vander Stoep A, Rao D, Kumar M. Risk factors for postpartum depression in women living with HIV attending prevention of mother-to-child transmission clinic at Kenyatta National Hospital, Nairobi. AIDS care. 2016;28(7):884–9. 10.1080/09540121.2016.1160026 27045273 PMC4965230

[30] Turner R, Honikman S. Maternal mental health and the first 1 000 days. SAMJ: South African Medical Journal. 2016;106(12):1164–7.

[31] Ickovics JR, Hamburger ME, Vlahov D, Schoenbaum EE, Schuman P, Boland RJ, et al Mortality, CD4 cell count decline, and depressive symptoms among HIV-seropositive women: longitudinal analysis from the HIV Epidemiology Research Study. Jama. 2001;285(11):1466–74. 11255423 10.1001/jama.285.11.1466

[32] Uwakwe R. Affective (depressive) morbidity in puerperal Nigerian women: validation of the Edinburgh Postnatal Depression Scale. Acta Psychiatrica Scandinavica. 2003;107(4):251–9. 12662247 10.1034/j.1600-0447.2003.02477.x

[33] Rubin LH, Cook JA, Grey DD, Weber K, Wells C, Golub ET, et al Perinatal depressive symptoms in HIV-infected versus HIV-uninfected women: a prospective study from preconception to postpartum. Journal of Women’s Health. 2011;20(9):1287–95. 10.1089/jwh.2010.2485 21732738 PMC3168970

[34] Sarason IG, Sarason BR, Shearin EN, Pierce GR. A brief measure of social support: Practical and theoretical implications. Journal of social and personal relationships. 1987;4(4):497–510.

[35] Baron EC, Davies T, Lund C. Validation of the 10-item centre for epidemiological studies depression scale (CES-D-10) in Zulu, Xhosa and Afrikaans populations in South Africa. BMC psychiatry. 2017;17(1):6 10.1186/s12888-016-1178-x 28068955 PMC5223549

[36] Mosack V, Shore ER. Screening for depression among pregnant and postpartum women. Journal of Community Health Nursing. 2006;23(1):37–47. 10.1207/s15327655jchn2301_4 16445363

[37] Andresen EM, Malmgren JA, Carter WB, Patrick DL. Screening for depression in well older adults: evaluation of. Prev Med. 1994;10:77–84. 8037935

[38] Zhang W, O’Brien N, Forrest JI, Salters KA, Patterson TL, Montaner JS, et al Validating a shortened depression scale (10 item CES-D) among HIV-positive people in British Columbia, Canada. PloS one. 2012;7(7):e40793 10.1371/journal.pone.0040793 22829885 PMC3400644

[39] Myer L, Smit J, Roux LL, Parker S, Stein DJ, Seedat S. Common mental disorders among HIV-infected individuals in South Africa: prevalence, predictors, and validation of brief psychiatric rating scales. AIDS patient care and STDs. 2008;22(2):147–58. 10.1089/apc.2007.0102 18260806

[40] Chishinga N, Kinyanda E, Weiss HA, Patel V, Ayles H, Seedat S. Validation of brief screening tools for depressive and alcohol use disorders among TB and HIV patients in primary care in Zambia. BMC psychiatry. 2011;11(1):75. 21542929 10.1186/1471-244X-11-75 PMC3112078

[41] Natamba BK, Achan J, Arbach A, Oyok TO, Ghosh S, Mehta S, et al Reliability and validity of the center for epidemiologic studies-depression scale in screening for depression among HIV-infected and -uninfected pregnant women attending antenatal services in northern Uganda: a cross-sectional study. BMC Psychiatry. 2014;14(1):303 10.1186/s12888-014-0303-y 25416286 PMC4260190

[42] Ramchandani PG, Richter LM, Stein A, Norris SA. Predictors of postnatal depression in an urban South African cohort. Journal of affective disorders. 2009;113(3):279–84. 10.1016/j.jad.2008.05.007 18571734

[43] Cox JL, Holden JM, Sagovsky R. Detection of postnatal depression: development of the 10-item Edinburgh Postnatal Depression Scale. The British journal of psychiatry. 1987;150(6):782–6. 3651732 10.1192/bjp.150.6.782

[44] Peltzer K, Rodriguez VJ, Lee TK, Jones D. Prevalence of prenatal and postpartum depression and associated factors among HIV-infected women in public primary care in rural South Africa: a longitudinal study. AIDS care. 2018:1–8. 10.1080/09540121.2018.1455960 PMC6150830 29575943

[45] Hugo CJ, Boshoff DE, Traut A, Zungu-Dirwayi N, Stein DJ. Community attitudes toward and knowledge of mental illness in South Africa. Social psychiatry and psychiatric epidemiology. 2003;38(12):715–9. 10.1007/s00127-003-0695-3 14689176

[46] Evagorou O, Arvaniti A, Samakouri M. Cross-cultural approach of postpartum depression: manifestation, practices applied, risk factors and therapeutic interventions. Psychiatric Quarterly. 2016;87(1):129–54. 10.1007/s11126-015-9367-1 25986531

[47] Holt C, Milgrom J, Gemmill AW. Improving help-seeking for postnatal depression and anxiety: a cluster randomised controlled trial of motivational interviewing. Archives of Women’s Mental Health. 2017:1–11. 10.1007/s00737-017-0767-0 28776105

[48] Kwalombota M. The effect of pregnancy in HIV-infected women. AIDS care. 2002;14(3):431–3. 10.1080/09540120220123829 12042089

[49] Barron P, Pillay Y, Doherty T, Sherman G, Jackson D, Bhardwaj S, et al Eliminating mother-to-child HIV transmission in South Africa. Bulletin of the World Health Organization. 2013;91:70–4. 10.2471/BLT.12.106807 23397353 PMC3537246

[50] Bor J, Herbst AJ, Newell M-L, Bärnighausen T. Increases in adult life expectancy in rural South Africa: valuing the scale-up of HIV treatment. Science. 2013;339(6122):961–5. 10.1126/science.1230413 23430655 PMC3860268

[51] Joint United Nations Programme on HIV/AIDS. A progress report on the global plan towards the elimination of new HIV infections among children by 2015 and keeping their mothers alive. 2013.

[52] Hutton HE, Lyketsos CG, Zenilman JM, Thompson RE, Erbelding EJ. Depression and HIV risk behaviors among patients in a sexually transmitted disease clinic. American Journal of Psychiatry. 2004;161(5):912–4. 10.1176/appi.ajp.161.5.912 15121659

[53] Rahman A, Iqbal Z, Harrington R. Life events, social support and depression in childbirth: perspectives from a rural community in the developing world. Psychological medicine. 2003;33(7):1161–7. 14580070 10.1017/s0033291703008286

[54] Taylor AL. Social Support: A Predictor of Postpartum Depression among HIV-positive and HIV-negative Women: Drexel University; 2012.

